# RINT1 Loss Impairs Retinogenesis Through TRP53-Mediated Apoptosis

**DOI:** 10.3389/fcell.2020.00711

**Published:** 2020-07-30

**Authors:** Anielle L. Gomes, Gabriel E. Matos-Rodrigues, Pierre-Olivier Frappart, Rodrigo A. P. Martins

**Affiliations:** ^1^Programa de Biologia Celular e do Desenvolvimento, Instituto de Ciências Biomédicas, Universidade Federal do Rio de Janeiro, Rio de Janeiro, Brazil; ^2^Institute of Toxicology, University Medical Center of the Johannes Gutenberg University Mainz, Mainz, Germany

**Keywords:** DNA damage response, replicative stress, neurodegeneration, visual system development, neurogenesis, ganglion cells, optic nerve hypoplasia

## Abstract

Genomic instability in the central nervous system (CNS) is associated with defective neurodevelopment and neurodegeneration. Congenital human syndromes that affect the CNS development originate from mutations in genes of the DNA damage response (DDR) pathways. RINT1 (*Rad50-interacting protein 1*) is a partner of RAD50, that participates in the cellular responses to DNA double-strand breaks (DSB). Recently, we showed that *Rint1* regulates cell survival in the developing brain and its loss led to premature lethality associated with genomic stability. To bypass the lethality of *Rint1* inactivation in the embryonic brain and better understand the roles of RINT1 in CNS development, we conditionally inactivated *Rint1* in retinal progenitor cells (RPCs) during embryogenesis. *Rint1* loss led to accumulation of endogenous DNA damage, but RINT1 was not necessary for the cell cycle checkpoint activation in these neural progenitor cells. As a consequence, proliferating progenitors and postmitotic neurons underwent apoptosis causing defective neurogenesis of retinal ganglion cells, malformation of the optic nerve and blindness. Notably, inactivation of *Trp53* prevented apoptosis of the RPCs and rescued the generation of retinal neurons and vision loss. Together, these results revealed an essential role for TRP53-mediated apoptosis in the malformations of the visual system caused by RINT1 loss and suggests that defective responses to DNA damage drive retinal malformations.

## Introduction

Several human diseases that affect the central nervous system (CNS) originate from mutations in genes of the DNA damage response (DDR) pathways (Jackson and Bartek, 2009; McKinnon, 2017). RINT1 (*Rad50-interacting protein 1*) was initially described as a regulator of the G2/M cell cycle checkpoint, centrosome integrity and chromosomal segregation (Xiao et al., 2001; Lin et al., 2007). Additional roles for RINT1 were described, including regulation of autophagy and Golgi-ER trafficking mechanisms (Hirose et al., 2004; Arasaki et al., 2006; He et al., 2014). *Rint1* inactivation in the developing brain is lethal, causes massive apoptosis of neural progenitor cells, and was associated with DNA damage accumulation, impaired ER-Golgi homeostasis and autophagy inhibition (Grigaravicius et al., 2016). While these findings reinforced the importance of RINT1 for progenitor cells survival, it remains unclear how and which of the multiple functions of RINT1 contributes to its pleiotropic effects in physiological and pathological contexts.

The neural retina is the CNS tissue that detects and transmits visual stimuli to the brain through axonal projections of the retinal ganglion cells that compose the optic nerve (Horsburgh and Sefton, 1986; Dowling, 1987). Malformation and/or degeneration of retinal ganglion cells can cause irreversible blindness (Taylor, 2007; Almasieh et al., 2012). The architecture of retinal tissue and the mechanisms that govern the generation of retina neurons during development are highly conserved in vertebrates, making the retina an excellent system to study neurogenesis in the CNS (Centanin and Wittbrodt, 2014). Retinal ganglion cells are the first neurons generated and, as well as other retinal cell types, originate from multipotent retinal progenitor cells (RPCs). Precise coordination of the RPCs proliferation, survival and neurogenesis is essential for the formation of a functional retina (Dyer and Cepko, 2001; Ohnuma and Harris, 2003) and it is well established that RPCs rely on classical cell cycle checkpoints in response to exogenous DNA damaging agents (Herzog et al., 1998; Borges et al., 2004; Mayer et al., 2016). However, few studies approached how defects in physiological DDR affects the genesis of retinal neurons (Baranes et al., 2009; Baleriola et al., 2010; Rodrigues et al., 2013; Alvarez-Lindo et al., 2019).

In humans, RINT1 mutations have been recently associated with a developmental multisystem disorder (Cousin et al., 2019) and in mice, loss of RINT1 *in vivo* causes progenitor cell death and is lethal (Lin et al., 2007; Grigaravicius et al., 2016). In a context where different molecular mechanisms for RINT1 have been described (Kong et al., 2006; Lin et al., 2007; Arasaki et al., 2013; Tagaya et al., 2014), characterizing how *Rint1* loss of function leads to cell death will contribute to determine its essential roles for progenitor homeostasis. TP53 is a master regulator of DDR and key for DNA damage induced cell death of progenitor cells, however TP53-independent responses to DNA damage have been reported (Pietsch et al., 2008; Valentine et al., 2011; Reinhardt and Schumacher, 2012; Fagan-Solis et al., 2020). Importantly, activation of DDR in the CNS of mice may trigger distinct TRP53-dependent outcomes (Frappart and McKinnon, 2007; Lee et al., 2012b; Lang et al., 2016), and it has not yet been studied whether TRP53 is required for the developmental malformations caused by RINT1 loss.

To bypass the lethality caused by *Rint1* inactivation in the embryonic brain and understand the long-term consequences of its inactivation to CNS development, we conditionally inactivated *Rint1* in retinal progenitor cells (RPCs). Our findings indicate that RINT1 is essential to prevent endogenous DNA damage accumulation, but is not required for the activation of cell cycle checkpoint. In *Rint1*-deficient retinas, RPC committed to differentiate into retinal ganglion cells die by apoptosis severely compromising retinogenesis and optic nerve formation. Remarkably, inactivation of *Trp53* in the *Rint1*-deficient retinas rescued the RPCs death and fully restored retinal structure and vision, demonstrating that RINT1is essential for retinal development and indicating that the cell death of progenitors is key for developmental malformations caused by RINT1 deficiency.

## Materials and Methods

### Ethics Statement, Mice, and Genotyping

All experiments with rodents were planned according to international rules and were approved by the Ethics Committee on Animal Experimentation of the Health Sciences Center (CEUA, CCS) of the Federal University of Rio de Janeiro in Brazil and approved by the governmental review board of the state of Baden-Württemberg (Regierungspräsidium Karlsruhe-Abteilung 3-Landwirtschaft, Ländlicher Raum, Veterinär-und Lebensmittelwesen) in Germany.

Transgenic mice lines used in this work: α-Cre (Tg(Pax6-cre,GFP)2Pgr) (Marquardt et al., 2001), Rint1 Flox (*Rint1*^TM 1^.^1Pof^) (Grigaravicius et al., 2016) and Trp53 Flox (B6.129P2-Trp53tm2Brn/A) (Jonkers et al., 2001). Mice were identified as follows: 1- control: α-Cre^–/–^; Rint1^Flox/Flox^ = = *Rint1*^*Ctrl*^; 2- cKO: α-Cre^+/–^; Rint1^Flox/Flox^ = *Rint1^α–*Cre*^*; 3- DKO: α-Cre^+/–^; Rint1^Flox/Flox^; Trp53^Flox/Flox^ = = *Rint1; Trp53^α–*Cre*^*. These transgenic mice were genotyped as described in the original publications: Rint1^Flox^ primers: Rint6956F (5′-AGTTCCTACTGACTTG CTGTGATAG-3′) and Rint7732R (5′-GTCAGGCCACAGAT TAGGCT-3′); Trp53^Flox^ primers: oIMR8543F (5′-GGTTAA ACCCAGCTTGACCAG-3′) and oIMR8544R (5′-GGAGGCA GAGACAGTTGGAG-3′). Cre-mediated recombination of the Rint1^Flox^ allele was verified using Rint6542F (5′-TAACCCCTG ACCCATCTCTC-3′) and Rint-8345R: (5′-ACTTCTGGATGA CTGAGGAC-3′) primers.

### RNA Extraction, cDNA Synthesis, and Real-Time RT-PCR

Retinas were dissected in cold PBS and lysed in 1 mL of Trizol (Thermo Fisher Scientific, cat# 15596026). Following, mechanical lysis of the tissue using a 100U syringe, standard Trizol extraction was performed and the pellet resuspended in 20 μL of ultrapure water (Thermo Fisher Scientific, 10977). Analysis of rRNA integrity was performed by electrophoresis in a 1% agarose gel and RNA concentration and purity were determined using a Nanodrop^TM^ 2000 spectrophotometer; 1 μg of total RNA was treated with DNase (rDNase kit, Ambion, AM1906) and contamination with genomic DNA was verified by PCR using primers for genomic DNA and electrophoresis. cDNA was synthetized using first-strand cDNA synthesis kit (GE, 27-9261-01) following the manufacturer’s instructions.

Real-time RT-PCR reactions were performed in an Applied Biosystems ABI7500 thermocycler. TaqMan and SYBR methods were used. Primers used for real-time RT-PCR: Rint1 forward 5′-GCGCTCCTTTCCTATGTGTCTG-3′, Rint1 reverse 5′-AGCC CTGGATGGATGACCTTGG-3′. TaqMan primers and probes: β-actin forward 5′-AGCCACCCCCACTCCTAAGA-3′; reverse 5′-TAATTTACACAGAAGCAATGCTGTCA-3′; probe 5′-ATGG TCGCGTCCATGCCCTGA-3′. For SYBR green (Applied Biosystems, 4367659), reactions had 12.5 μL of SYBR Green 2× mix, 2 μL of diluted cDNA (1:10), 0.5 μL (5 μM) of each primer and 9.5 μL of UltraPure water (Gibco, 10977). For TaqMan (Thermo Fisher Scientific, 4369016), reactions had 10 μL of 2× TaqMan mix, 1 μL of diluted cDNA (1:10), 0.4 μL (5 μM) of each primer, 0.2 μL of probe (5 μM) and 8 μL of UltraPure water. The cycling conditions were: 50°C for 2 min, 95°C for 10 min and 40 cycles of 94°C for 15 s and 60°C for 60 s. Each sample was reacted in duplicate, and only duplicates with <0.5 Ct variation were further analyzed. The comparative method for relative quantification delta–delta Ct (2-ΔΔCt) was applied to determine the relative quantity of a target compared to the average of the reference gene (β-actin). We used a mathematical correction similar to the qBASE software based on the use of the mean of the ΔCt of all groups to define the value calibrator (Hellemans et al., 2007).

### Immunostaining, TUNEL Assay, and Pyknotic Nuclei Identification

Eyes were fixed by immersion in 4% paraformaldehyde in PBS for 16 h, washed in PBS and cryoprotected in increasing concentrations of sucrose (10, 20, and 30% - 16 h each). Cryoprotected eyes were embedded in OCT, cut in a cryostat (Leica CM1850) and transversal sections (10 μm) were mounted on poly-L-lysine (300 μg/mL) covered slides. These were washed with PBS and antigen retrieval was performed (1-min boil in 10 mM citrate buffer, pH = 6). Slides were incubated in a blocking solution [5% goat serum (Sigma, cat# G9023); 1% bovine serum albumin (Sigma, cat# A2153); 0.5% Triton (Sigma, cat# X100)] for 30 min. All primary antibodies were diluted in blocking solution and incubated for 16 h at 4°C in the following dilutions: anti-Ser10 pH3 (1:200, CST, cat# 9701), anti-active caspase-3 (1:100, BD Biosciences, cat# 559565), anti-γH2AX (1:250, Millipore, cat# 05-636), anti-BrdU (1:3, GE, cat# RPN20), anti-Atoh7 (1:300, Novus, cat# 88639), and PCNA (1:400, SC, cat# SC-56). Immunofluorescence reactions were performed by different methods: biotin conjugated secondary antibody followed by the incubation with Cy3-conjugated streptavidin (red staining) (Thermo Fisher Scientific, cat# 434315) or an Alexa secondary antibody (green staining) (1:500, Life, cat# A11001 or A11008). Fluorescent nuclear counterstaining was performed using DAPI (Lonza, cat# PA3013) or Sytox Green (Thermo Fisher Scientific, cat# S7020).

To label S-phase cells *in vivo*, intraperitoneal injections of 50 μg/g of body weight of BrdU (Sigma Aldrich, cat# B5002) were performed. Eyes were collected 1 h after injection. TUNEL [Click-iT TUNEL Alexa Fluor 488 Imaging Assay (Invitrogen, C10245)] analysis was performed following manufacturer’s instructions. Fluorescent images were captured using a Leica TCS-SPE with an AOBS confocal microscope system. In addition to TUNEL assay and cleaved-caspase-3 staining, apoptotic cell death was also analyzed through the detection of pyknotic nuclei, a classical morphological hallmark of apoptosis. Pyknotic nuclei were identified in retinal tissue sections previously stained with nuclear dyes (DAPI or SYTOX green) based on its morphology of compacted, spherical and intense (brighter) nuclear staining that reveals the higher degree of nuclear chromatin condensation (Soriano et al., 1993; Ziegler and Groscurth, 2004; Kroemer et al., 2009) (Figure 3A).

### Optomotor Response Test

Measurements of visual acuity by optomotor response were performed using OptoMotry as previously described (Cavalheiro et al., 2017; Rocha-Martins et al., 2019). Visual accuracy threshold was determined by systematic increments of the spatial frequency until the animal no longer responded. The experimenter was blind in relation to mice genotypes.

### Experimental Design, Quantifications, and Statistical Analysis

At least three mice were used on each analysis and the number of mice used on each experiment was plotted as a dot in each graph (black dots for control = *Rint1*^*Ctrl*^, brown dots for cKO = *Rint1^α–*Cre*^* and red dots for DKO = *Rint1; Trp53^α–*Cre*^* mice. For every statistical analysis, the measurement obtained for each mouse in a given experiment was used as an independent value (*n*). Due to the pattern of the Cre-mediated recombination in α-Cre retinas (Marquardt et al., 2001) (Cre recombination occurs only in retinal periphery), in experiments involving histological sections, we analyzed and quantified only the retinal periphery (∼250 micrometers most-peripheral regions of each side of the retinal section). To standardize regions between different samples, only sections in which the optic nerve was visible were used for quantifications. At least three sections from each mouse were quantified and the obtained mean was the measurement used for each mouse. Quantifications in the neuroblastic layer (NBL) were normalized by area (mm^2^) and quantifications on the ganglion cell layer (GCL) were normalized by length (μm) of retinal tissue. GraphPad Prism software was used for statistical analysis. Student’s *t*-test or one-way ANOVA were performed as indicated on each figure legend. Computations assumed the same scatter (s.d.) and Gaussian distribution between groups. *p*-values are based on two-sided tests.

## Results

### RINT1 Is Essential for Retinal Development and Its Loss Causes Blindness

**FIGURE 1:**
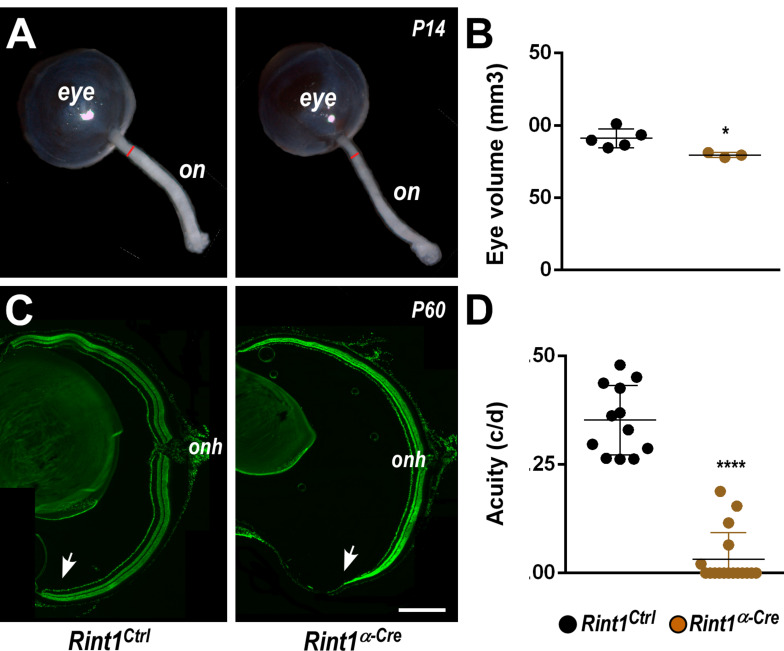
Retinal progenitor cell-specific inactivation of *Rint1* severely impairs retinogenesis causing blindness. **(A)** Representative images of optic nerve and **(B)** eye volume measurements of *Rint1*^*Ctrl*^ and *Rint1^α–*Cre*^* mice at P14. **(C)** Representative images of the *Rint1*^*Ctrl*^ and *Rint1^α–*Cre*^* retinal sections stained with SYTOX green at P60. **(D)** Behavioral optomotor response analysis in the *Rint1*^*Ctrl*^ and *Rint1^α–*Cre*^* mice at 4 months. Statistical analysis: **(A)** Student’s *t*-test; **(D)** One-way ANOVA followed by Tukey’s post-test; *p < 0.05; ****p < 0.0001. Error bars indicate SD. Scale bar: 1 mm. On, optic nerve; Onh, optic nerve head; c/d, cycles/degree.

To investigate RINT1 function during retinogenesis, we used a previously generated *Rint1* floxed mice (Grigaravicius et al., 2016) and crossed with an α-Cre mouse line (Marquardt et al., 2001) that leads to *Rint1* genetic inactivation in retinal progenitor cells (RPCs). Real-time RT-PCR studies revealed that *Rint1* is expressed through out mouse retinal development (Supplementary Figure S1A) and PCR analysis confirmed the recombination of the floxed allele in the *Rint1^α–*Cre*^* (*Rint1^*F/F*^;* α*-Cre^+/–^*) retina (Supplementary Figure S1B). Inactivation of *Rint1* specifically in the RPCs induced optic nerve hypoplasia and mildly affected eye growth (Figures 1A,B). Consistent with the spatial pattern of α-Cre-mediated recombination (Marquardt et al., 2001), the periphery of adult *Rint1*-deficient retinas was severely affected, confirming that RINT1 is required for retinal morphogenesis (Figure 1C). To test whether the malformation of *Rint1*-deficient retinas would impact visual function, we performed an optomotor response analysis that revealed a severe visual acuity impairment of *Rint1^α–*Cre*^* mice (Figure 1D). These findings indicate that RINT1 is crucial for retinal development and for visual function.

### DNA Damage Accumulation and Checkpoint Activation Following RINT1 Loss

**FIGURE 2:**
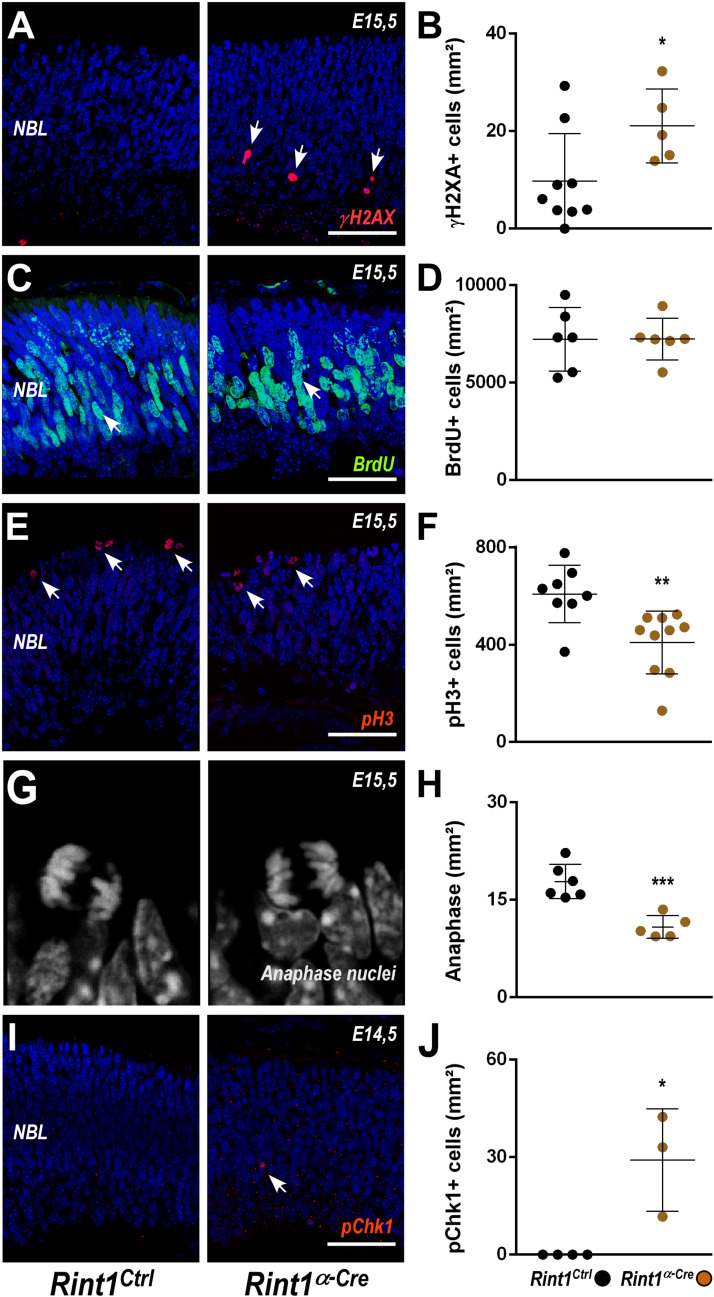
DNA damage accumulation and normal cell cycle checkpoint following *Rint1* inactivation in RPCs. **(A,C,E,G,I)** Representative images of γH2AX, BrdU, phospho-H3 (pH3), anaphase mitotic nuclei, and phospho-Chk1 (pChk1) immunostaining in *Rint1*^*Ctrl*^ and *Rint1^α–*Cre*^* retinas at E14.5 or E15.5 (as indicated). **(B,D,F,H,J)** Quantification of γH2AX^+^, BrdU^+^, pH3^+^, anaphase nuclei, and pChk1^+^ cells in *Rint1*^*Ctrl*^ and *Rint1^α–*Cre*^* retinas at E14.5 or E15.5. Statistical analysis: Student’s *t*-test. *p < 0.05; **p < 0.01; ***p < 0.001. Error bars indicate SD. Scale bars: 50 μm. NBL, neuroblastic layer.

To better understand the defective morphogenesis of *Rint1^α–*Cre*^* retina, we evaluated the consequences of RINT1 loss to key cellular events of early retinogenesis. In progenitor cells of the brain, *Rint1* inactivation caused genomic instability (Grigaravicius et al., 2016); therefore, we asked whether RINT1 loss would affect the DDR in RPCs. An increased proportion of γH2AX positive (+) cells suggested an accumulation of endogenous DNA damage in the *Rint1*-deficient RPCs (Figures 2A,B). Since DNA damage can activate distinct cell cycle checkpoints and pause the cell cycle, we asked whether the proliferation of RPCs would be affected following RINT1 loss. First, we analyzed the distribution and scored the proportion of PCNA, a progenitor cell marker expressed in all phases of the cell cycle. No difference in PCNA^+^ cells was found in the *Rint1^α–*Cre*^* embryonic retinas (E15.5) (Supplementary Figure S2). Next, we pulse-labeled progenitor cells entering the S-phase with bromodeoxyuridine (BrdU) and quantified the proportion of BrdU^+^ RPCs. No alteration in the proportion of BrdU^+^ cells was observed (Figures 2C,D), indicating that total number of RPCs is unaltered and that these progenitors normally enter S-phase in *Rint1*-deficient retinas. RINT1 was previously associated with the regulation of G2/M cell cycle checkpoint following irradiation (Xiao et al., 2001). To test whether inactivation of *Rint1* could impact the transition of progenitors between cell cycle phases, we scored phospho-histone H3 (pH3)^+^ RPCs and, based on the nuclear morphology, the number of RPCs reaching anaphase. A decrease in pH3^+^ cells (Figures 2E,F) and a reduction of RPCs in anaphase (Figures 2G,H) was detected in the *Rint1^α–*Cre*^* retinas, suggesting that the accumulation of DNA damage caused by RINT1 loss activates a cell cycle checkpoint that prevents RPCs to reach final phases of mitosis.

**FIGURE 3:**
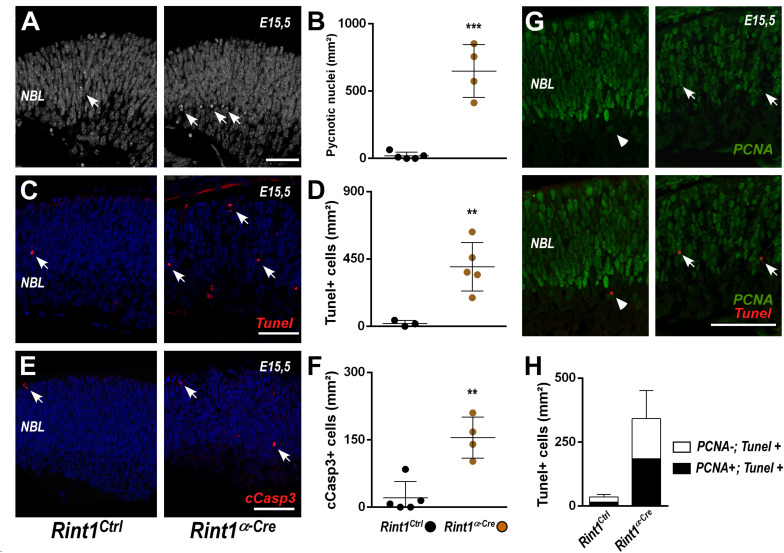
RINT1 loss induces apoptosis of progenitors and postmitotic retinal cells. **(A,C,E)** Representative images of pyknotic nuclei (DAPI staining in **A**), TUNEL, cleaved-caspase 3 (cCasp3) immunostaining in *Rint1*^*Ctrl*^ and *Rint1^α–*Cre*^* retinas at E15.5. **(B,D,F)** Quantification of pyknotic nuclei, TUNEL^+^, and cCasp3^+^ cells in *Rint1*^*Ctrl*^ and *Rint1^α–*Cre*^* retinas at E15.5. **(G,H)** Representative images of PCNA and TUNEL double staining and quantification of PCNA^+^ (arrow) and PCNA-negative (arrowhead) cells among TUNEL^+^ in *Rint1*^*Ctrl*^ and *Rint1^α–*Cre*^* retinas at E15.5. Statistical analysis: Student’s *t*-test. **p < 0.01; ***p < 0.001. Error bars indicate SD. Scale bars: 50 μm. NBL, neuroblastic layer.

ATR-mediated phosphorylation of Chk1 is a hallmark of replicative stress and mediates both intra-S and G2/M checkpoints (Liu et al., 2000; Saldivar et al., 2018). To test whether RINT1 loss would lead to Chk1 activation in RPCs, we scored the proportion of phospho-Chk1 (pChk1)^+^ cells. An increase of pChk1^+^ cells was observed in *Rint1^α–*Cre*^* embryonic retinas (Figures 2I,J). Altogether, these findings indicate that in the absence of RINT1, RPCs accumulate endogenous DNA damage, likely during replication, and activate cell cycle checkpoints in the absence of RINT1.

### *Rint1* Inactivation Induces Cell Death in the Embryonic Retina

Replication-associated accumulation of DNA damage and activation of cell cycle checkpoints may induce cell death (Nowsheen and Yang, 2012; Saldivar et al., 2017), therefore we interrogated whether RINT1 loss would cause cell death in developing retina. An increase in apoptosis was observed in *Rint1*-deficient embryonic retinas as revealed by the quantification of pyknotic nuclei (Figures 3A,B), TUNEL^+^ (Figures 3C,D) and cleaved caspase-3 (cCasp3^+^) cells (Figures 3E,F). During mid-gestational stages of mouse retinogenesis, in addition to the expansion of progenitor pools, a proportion of the RPCs exit cell cycle and undergo cell differentiation (Agathocleous and Harris, 2009). To determine whether RINT1 loss would induce apoptotic cell death of RPCs, we performed a double staining for TUNEL and PCNA at E15.5. Approximately half of the TUNEL^+^ cells were PCNA^+^ in *Rint1^α–*Cre*^* retinas (Figures 3G,H), confirming that proliferating RPCs undergo apoptosis and suggesting that postmitotic cells may also die following *Rint1* inactivation.

### Apoptosis of *Rint1*-Deficient RPCs Compromises Ganglion Cell Layer Generation

**FIGURE 4:**
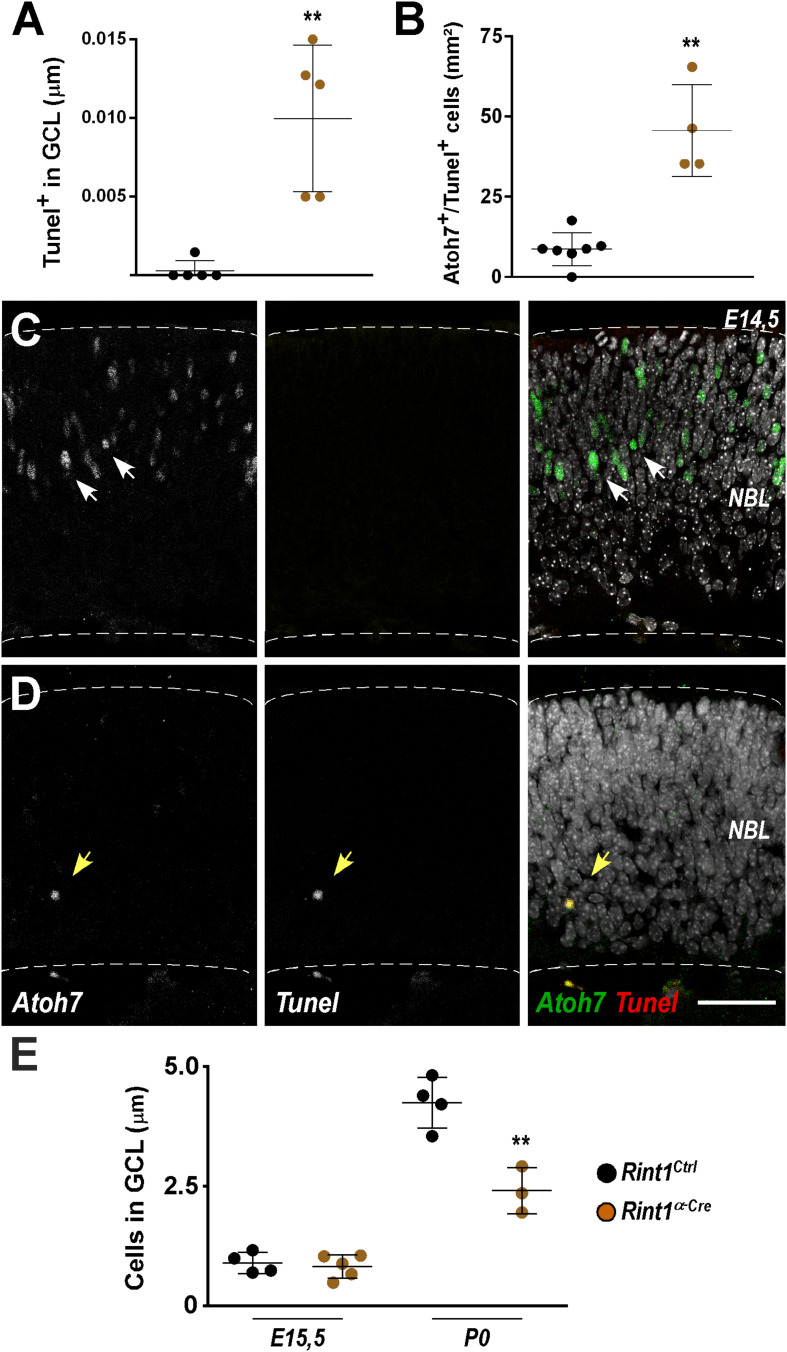
Defective genesis of retinal ganglion cell layer in *Rint1*-deficient retinas. **(A)** Quantification of TUNEL^+^ cells in the GCL of *Rint1*^*Ctrl*^ and *Rint1^α–*Cre*^* at E15.5. **(B–D)** Quantification of Athonal 7^+^ (*Atoh7*) and TUNEL^+^ cells, and representative images of Atoh7 and TUNEL double staining in *Rint1*^*Ctrl*^ **(C)** and *Rint1^α–*Cre*^* **(D)** retinas at E14.5. **(E)** Quantification of the total number of cells in the ganglion cell layer (GCL) in *Rint1*^*Ctrl*^ and *Rint1^α–*Cre*^* retinas at E15.5 and postnatal day 0 (P0). Statistical analysis: Student’s *t*-test. **p < 0.01. Error bars indicate SD. Scale bar: 50 μm. NBL, neuroblastic layer.

Retinal ganglion cells are the first cell type to be generated during retinogenesis (Sidman, 1961; Rapaport et al., 2004). In the mouse, their birth begins around E11, peaks during mid-gestation while newborn retinal ganglion cells migrate to the ganglion cell layer (GCL) (Drager, 1985; Young, 1985; Nguyen-Ba-Charvet and Rebsam, 2020). The detection of PCNA-negative apoptotic cells in *Rint1*-deficient retinas may be explained by the loss of PCNA in dying progenitors or by the apoptosis of postmitotic cells after RINT1 loss. Therefore, we tested the hypothesis that *Rint1*-deficiency affects RPCs committed to become ganglion cells and/or postmitotic cells that migrate toward the GCL. Quantification of TUNEL^+^ cells in the GCL confirmed that postmitotic neurons die in *Rint1*-deficient embryonic retinas (Figure 4A). To examine whether RINT1 loss affects RPCs committed to differentiate into retinal ganglion cells, we performed a double staining for TUNEL and Athonal 7 (Atoh7), a master regulator of retinal ganglion cells identity and differentiation (Brown et al., 2001; Wang et al., 2001; Yang et al., 2003; Brzezinski et al., 2012). The proportion of TUNEL/Atoh7 double positive RPCs sharply increased in *Rint1^α–*Cre*^* retinas (Figures 4B–D). Next, we asked whether the apoptosis of postmitotic neurons and of RPCs committed to become ganglion cells in *Rint1^α–*Cre*^* retina affect the formation of the GCL, where ganglion cells and displaced amacrine cells reside after migration. No alteration in the number of neurons in the GCL was detected at E15.5; however, during postnatal stages, fewer neurons occupy the GCL of *Rint1^α–*Cre*^* retinas (Figure 4E). These findings suggest that the defective neurogenesis and optic nerve hypoplasia of *Rint1^α–*Cre*^* mice is caused by the apoptosis of both postmitotic neurons and committed RPCs.

### Trp53 Inactivation Rescues Phenotypes Caused by RINT1 Loss

**FIGURE 5:**
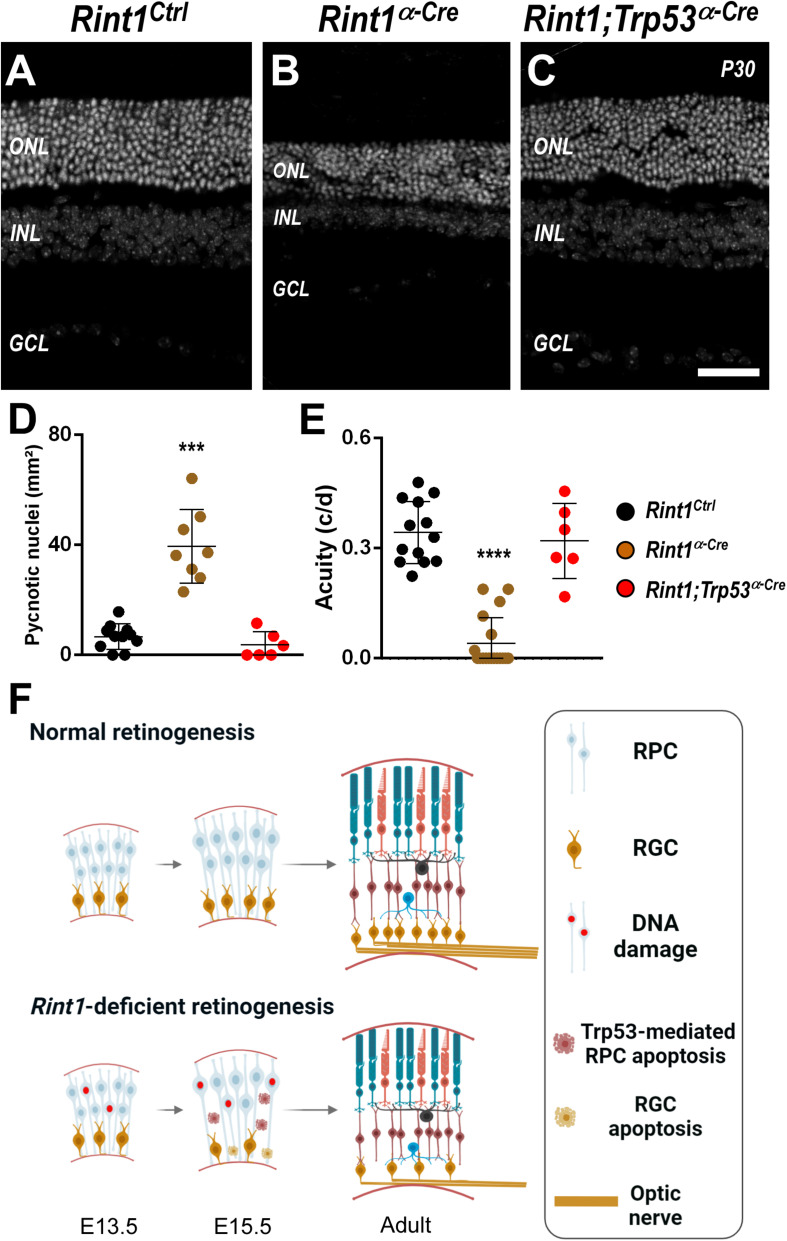
*Trp53* inactivation rescues apoptosis, morphological defects, and visual impairment caused by RINT1 loss. **(A–C)** Representative images *Rint1*^*Ctrl*^, *Rint1^α–*Cre*^*, and *Rint1;Trp53^α–*Cre*^* retinal tissue sections stained with DAPI (P30). **(D)** Quantification of the pyknotic nuclei in *Rint1*^*Ctrl*^, *Rint1^α–*Cre*^*, and *Rint1;Trp53^α–*Cre*^* retina at P0. **(E)** Behavioral optomotor response analysis in *Rint1*^*Ctrl*^, *Rint1^α–*Cre*^*, and *Rint1;Trp53^α–*Cre*^* mice at 4 months. **(F)** We propose a model in which RINT1 regulates DNA damage accumulation in RPCs. Its loss leads to *Trp53*-mediated apoptosis that impairs the generation of retinal ganglion cells and drives retinal malformations. Statistical analysis: One-way ANOVA followed by Tukey’s post-test. ***p < 0.001; ****p < 0.0001. Scale bar: 100 μm. c/d: cycles/degree.

Whenever *Rint1* was inactivated *in vivo* progenitor cells died causing severe phenotypes (Lin et al., 2007; Grigaravicius et al., 2016). Inactivation of DDR and DNA repair factors in neural progenitors lead to DNA damage-induced TRP53-dependent apoptosis (Frappart and McKinnon, 2007; Lee et al., 2012b). Previously, Grigaravicius et al. found evidence of TRP53 stabilization in *Rint1*-deficient neural progenitor cells, but the role of TRP53 was not studied. Therefore, to test whether TRP53-mediated apoptosis drives the malformations of *Rint1*-deficient retinas, we generated a *Rint1; Trp53^α–*Cre*^* mice DKO. Adult DKO retinas displayed all nuclear and plexiform layers and phenotypically resemble control retinas, indicating that *Trp53* inactivation fully rescued the retinogenesis of *Rint1*-deficient retinas (Figures 5A–C). Quantification of pyknotic nuclei revealed that *Trp53* inactivation prevented the apoptosis caused by RINT1 loss in developing retinas (Figure 5D). Finally, the DKO mice displayed a normal optomotor response, confirming that blockade of RPCs apoptosis fully rescued retina morphology and vision (Figure 5E). These findings indicate that the TRP53-mediated cell death of the *Rint1*-deficient neural progenitor cells drives the defective morphogenesis caused by RINT1 loss in the CNS (Figure 5F).

## Discussion

Visual function relies on the coordination of progenitor cells expansion and neurogenesis during retinal development. The comprehension of the molecular basis of how physiological DNA damage affects retinogenesis is still limited and may have relevant implications for regenerative medicine. Here, we showed that RINT1 protects retinal progenitor cells against DNA damage and apoptosis *in vivo*. In the absence of RINT1, retinogenesis was severely affected, leading to optic nerve malformation and vision impairment as revealed by optomotor response tests. Our model of retina-specific inactivation of *Rint1* suggests that retina structure and electrical function are compromised. However, further functional analysis, such as electroretinogram (e.g., flash visual evoked potentials – VEP) or pattern VEP are required to determine the exact functional deficits contributing to the decreased visual acuity. Our findings are summarized in Figure 5F.

Multiple cellular and molecular mechanisms were previously described for RINT1 (Xiao et al., 2001; Kong et al., 2006; Lin et al., 2007; Arasaki et al., 2013). In the brain, RINT1 prevents genomic instability, regulates ER/Golgi homeostasis and is required for the clearance of autophagosomes (Grigaravicius et al., 2016). Here, we show that shortly after RINT1 loss, progenitor cells committed to differentiate into ganglion cells accumulate DNA damage and undergo TRP53-mediated apoptosis. It was proposed that RINT1 and RAD50 interact and regulate G2/M cell cycle checkpoint in response to irradiation (Xiao et al., 2001), but little is known about how RINT1 prevents the accumulation of endogenous DNA damage in progenitor cells. In contrast to previous studies, our finding that fewer RPCs reached anaphase in *Rint1*-deficient retinas, indicate that RINT1 is not essential for the activation of functional cell cycle checkpoints in neural progenitor cells. The activation of ATR kinase in *Rint1*-deficient RPCs, as demonstrated by the phosphorylation of CHK1, suggests that DNA damage may arise during DNA replication. Indeed, RINT1 function is directly related to the MRN complex that is essential for the repair of DNA double strand breaks (Lamarche et al., 2010; Scully et al., 2019). More specifically, during DNA replication, the MRN complex participates in the activation of ATR, resolution of transcription–replication conflicts and replication fork restart (Duursma et al., 2013; Syed and Tainer, 2018). We hypothesize that RINT1 loss leads to replicative stress by disturbing the function of RAD50 and, thereafter, the MRN complex. In this context, we have shown that NBS1/Nbn also protects retinal progenitor cells from DNA damage and apoptosis, highlighting the importance of these pathways for neural progenitor cells homeostasis (Rodrigues et al., 2013). Studies about the mechanisms of RINT1 during replication may provide important insights of how neural progenitors control genome stability.

The consequences of defective DDR and its impact in developmental neurogenesis have been well studied in the brain. Inactivation of components of DNA replication machinery, DNA damage signaling pathways (Frappart et al., 2005; Lee et al., 2012a, b) as well as DNA repair factors (Lee et al., 2000; Frappart and McKinnon, 2007, 2008; Baranes et al., 2009) revealed different levels of CNS malformations. In contrast, even though congenital disorders caused by mutations in DDR genes exhibit retinal malformations (Lim and Wong, 1973; Erdöl et al., 2003; Bhisitkul and Rizen, 2004; Chai et al., 2009; Krzyżanowska-Berkowska et al., 2014; Sasoh et al., 2014), the impact of defective DDR in retinogenesis and visual impairment still awaits investigation. Studies about the DNA damage signaling and repair factors revealed optic nerve morphological alterations in *Nbn*-deficient retinas, but loss of NBN and ATM did not impact retinal neurogenesis (Baranes et al., 2009; Rodrigues et al., 2013). Consistent with the reduced cellularity of the ganglion cell layer, *Rint1*-deficient retinas also displayed malformation of the optic nerve. However, it cannot be discarded that defective axon growth or guidance may contribute to the described phenotype. In addition, because RINT1 loss impaired the generation of cells of the ganglion cell layer and possibly other cell types, perhaps RINT1 may have DDR-independent roles in the developing retina. Even though RINT1 was shown regulate ribosomal gene transcription (Yang et al., 2016), we do not anticipate a role of RINT1 in transcriptional networks of retinal cell types specification and propose that RPC apoptosis is a major driver of the retinal malformations. An interesting question in the field is why distinct DDR response pathways differentially affect neurogenesis. Considering that the retina is an ideal model to investigate neurogenesis, further studies may lead to a better comprehension of the relationship between DDR and neurogenesis with broad implications to the whole nervous system.

*Rint1* inactivation in non-dividing postmitotic neurons of the adult cerebellum causes neurodegeneration of Purkinje cells (Grigaravicius et al., 2016). During embryogenesis, RINT1 is essential for the survival of committed RPCs (Atoh7^+^) and postmitotic neurons of retinal ganglion cell layer (GCL). The apoptosis of retinal cell types that compose the GCL may be due to the previous accumulation of DNA damage in RPCs before they exit cell cycle. However, genomic instability independent functions of RINT1 in early-born retinal ganglion cells may not be ruled out. In *Rint1*-deficient cerebellum, 35% of Purkinje cell exhibited Golgi fragmentation while less than 1% accumulated DNA damage (Grigaravicius et al., 2016), suggesting that defective DDR may have a limited contribution to the degeneration of adult cerebellar neurons. ER-Golgi homeostasis, vesicle trafficking and autophagy were also shown to be important for the survival of retinal ganglion cells during retinogenesis and optic nerve degeneration (Boya et al., 2016; Adornetto et al., 2020). Further studies will be necessary to determine whether the apoptosis of postmitotic retinal neurons may be due to the previous accumulation of DNA damage in RPCs or pleiotropic RINT1 functions in these non-dividing neurons.

The relevance of RINT1 for human diseases was highlighted by several studies. The tumor predisposition of *Rint1* heterozygous mice indicated a role as tumor suppressor (Lin et al., 2007). Interestingly, genomic studies of human cancers suggested an oncogene or cancer predisposition gene function in glioblastomas, breast cancer and acute myeloid leukemia (Quayle et al., 2012; Park et al., 2014; Shahi et al., 2019; Simonetti et al., 2019). *RINT1* mutations were identified in patients of the ALF multisystem developmental disorder (Cousin et al., 2019) and in patients of Lynch syndrome (Park et al., 2014), that often presents retinal pigment epithelium hypertrophy (CHRPE) (Lynch et al., 1987). While, RINT1 variants may have the potential to impact protein-protein interactions (Otterpohl and Gould, 2017), the mechanisms underlying the contributions of RINT1 to these pathologies are not yet understood. TRP53-mediated cell cycle arrest and apoptosis are common responses to DNA damage in progenitor cells (Hafner et al., 2019). Because blockade of TRP53-mediated apoptosis fully rescued retina morphogenesis and function, we propose the cell death of progenitors is key for developmental malformations caused by RINT1 deficiency. Understanding the biology of that dictates accumulation of physiological DNA damage and progenitor cells elimination is of great importance a wide range of human pathological conditions, including developmental diseases and cancer.

## Data Availability Statement

All datasets presented in this study are included in the article/Supplementary Material.

## Ethics Statement

The animal study was reviewed and approved by the Ethics Committee on Animal Experimentation of the Health Sciences Center (CEUA, CCS) of the Federal University of Rio de Janeiro, Brazil, and Baden-Württemberg (Regierungspräsidium Karlsruhe-Abteilung 3-Landwirtschaft, Ländlicher Raum, Veterinär-und Lebensmittelwesen) in Germany.

## Author Contributions

AG, P-OF, and RM conceived and designed the experiments. AG, GM-R, and RM analyzed the data and performed the experiments. AG, GM-R, P-OF, and RM wrote the manuscript. All authors contributed to the article and approved the submitted version.

## Conflict of Interest

The authors declare that the research was conducted in the absence of any commercial or financial relationships that could be construed as a potential conflict of interest.

## Supplementary Material

FIGURE S1 *Rint1* expression and genetic inactivation in developing mouse retina. **(A)** Real-time RT-PCR for *Rint1* in the wild-type mouse retinas at E15.5, P0, P4, P9, P15, and P60. TaqMan probes for *Actb* were used as loading controls (*n* = 3). **(B)** PCR analysis of the *Rint1* allele recombination in P0 retinas.

FIGURE S2 **(A)** Representative images of PCNA immunostaining and **(B)** quantification of PCNA^+^ cells in *Rint1*^*Ctrl*^ and *Rint1^α–*Cre*^* retinas at E15.5. Scale bar: 50 μm.
