# Supplementary figures and images for: RINT1 Loss Impairs Retinogenesis Through TRP53-Mediated Apoptosis
Source: Front Cell Dev Biol. 2020 Jul 30;8:711. doi: 10.3389/fcell.2020.00711 (PMC7406574; doi:10.3389/fcell.2020.00711)

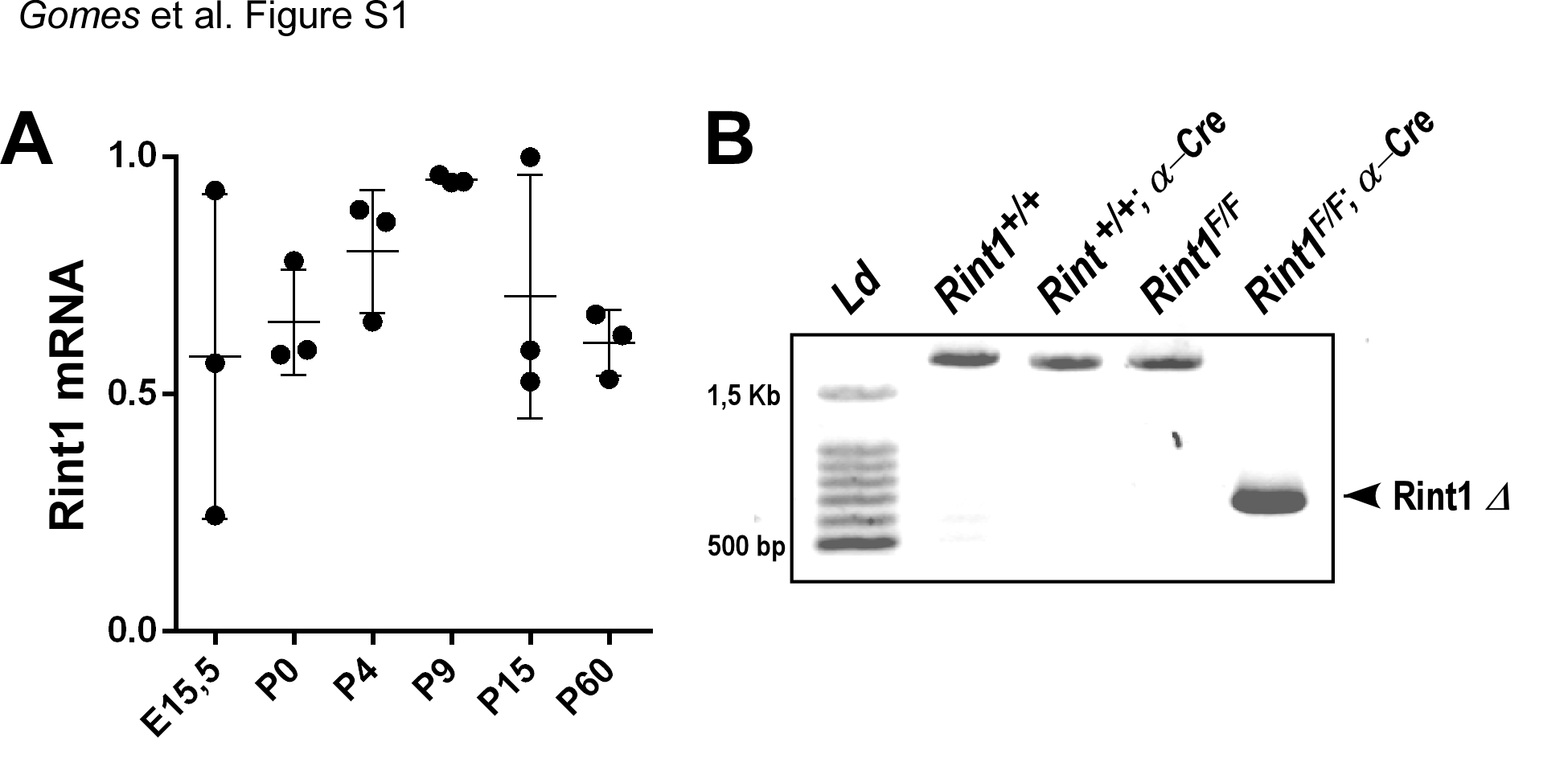

Supplement: FIGURE S1 — Rint1 expression and genetic inactivation in developing mouse retina. (A) Real-time RT-PCR for Rint1 in the wild-type mouse retinas at E15.5, P0, P4, P9, P15, and P60. TaqMan probes for Actb were used as loading controls (n = 3). (B) PCR analysis of the Rint1 allele recombination in P0 retinas. [file Image_1.TIF]

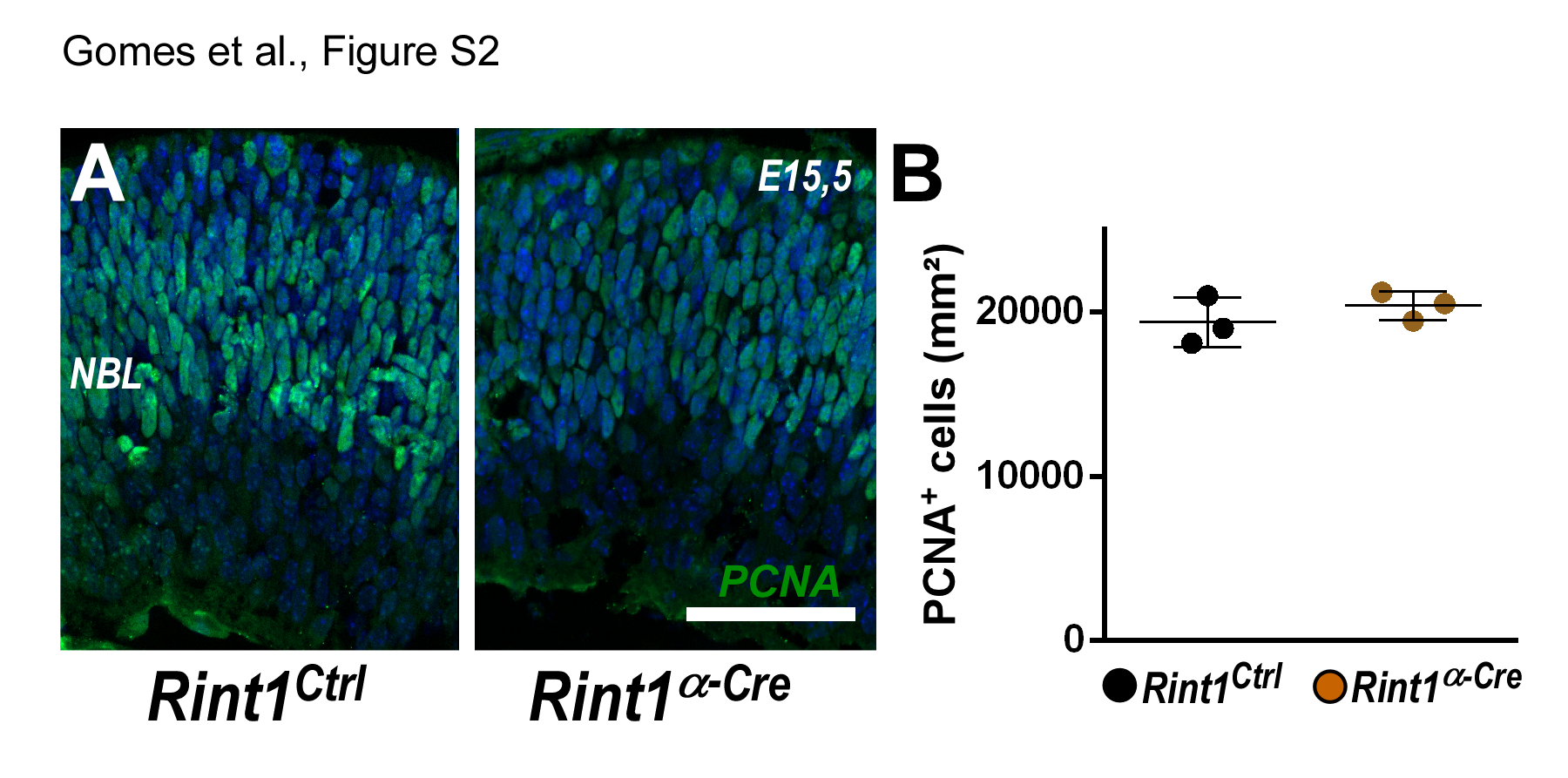

Supplement: FIGURE S2 — (A) Representative images of PCNA immunostaining and (B) quantification of PCNA+ cells in Rint1Ctrl and Rint1α–Cre retinas at E15.5. Scale bar: 50 μm. [file Image_2.TIF]
